# Dietary protein intake and mortality among survivors of liver cirrhosis: a prospective cohort study

**DOI:** 10.1186/s12876-023-02832-1

**Published:** 2023-07-03

**Authors:** Ghazal Daftari, Asal Neshatbini Tehrani, Fereshteh Pashayee-khamene, Sara Karimi, Saleheh Ahmadzadeh, Azita Hekmatdoost, Amin Salehpour, Mahdi Saber-Firoozi, Behzad Hatami, Zahra Yari

**Affiliations:** 1 School of Medicine, Tehran University of Medical Sciences, Tehran, Iran; 2 Student Research Committee, Ahvaz Jundishapur University of Medical Sciences, Ahvaz, Iran; 3 Department of Nutrition, School of Allied Medical Sciences, Ahvaz Jundishapur University of Medical Sciences, Ahvaz, Iran; 4 Clinical Nutrition and Dietetics Department, Faculty of Nutrition Sciences and Food Technology, National Nutrition and Food Technology Research Institute, Shahid Beheshti University of Medical Sciences Tehran, Tehran, Iran; 5 Occupational Health Research Center, School of Public Health, Iran University of Medical Science, Tehran, Iran; 6 Digestive Disease Research Center, Digestive Disease Research Institute, Tehran University of Medical Sciences, Tehran, Iran; 7 Gastroenterology and Liver Diseases Research Center, Research Institute for Gastroenterology and Liver Diseases, Shahid Beheshti University of Medical Sciences, Tehran, Iran; 8 Department of Nutrition Research, National Nutrition and Food Technology Research Institute and Faculty of Nutrition Sciences and Food Technology, Shahid Beheshti University of Medical Sciences Tehran, West Arghavan St. Farahzadi Blvd., Sharake Qods, Tehran, Iran

**Keywords:** Protein, Dairy, Cirrhosis, Mortality, Cohort study

## Abstract

**Background:**

Liver cirrhosis is a worldwide burden and is associated with poor clinical outcomes, including increased mortality. The beneficial effects of dietary modifications in reducing morbidity and mortality are inevitable.

**Aim:**

The current study aimed to evaluate the potential association of dietary protein intake with the cirrhosis-related mortality.

**Methods:**

In this cohort study, 121 ambulatory cirrhotic patients with at least 6 months of cirrhosis diagnosis were followed-up for 48 months. A 168-item validated food frequency questionnaire was used for dietary intake assessment. Total dietary protein was classified as dairy, vegetable and animal protein. We estimated crude and multivariable-adjusted hazard ratios (HRs) with 95% confidence intervals (CIs), applying Cox proportional hazard analyses.

**Results:**

After full adjustment for confounders, analyses showed that total (HR = 0.38, 95% CI = 0.2–1.1, p trend = 0.045) and dairy (HR = 0.38, 95% CI = 0.13–1.1, p trend = 0.046) protein intake was associated with a 62% lower risk of cirrhosis-related mortality. While a higher intake of animal protein was associated with a 3.8-fold increase in the risk of mortality in patients (HR = 3.8, 95% CI = 1.7–8.2, p trend = 0.035). Higher intake of vegetable protein was inversely but not significantly associated with mortality risk.

**Conclusion:**

A comprehensive evaluation of the associations of dietary protein intake with cirrhosis-related mortality indicated that a higher intakes of total and dairy protein and a lower intakes of animal protein are associated with a reduced risk of mortality in cirrhotic patients.

## Introduction

Liver cirrhosis is the first cause of liver-related mortality and morbidity globally with an annual exceeding mortality rate [1]. About one million deaths worldwide are reported every year due to cirrhosis which can be prevented [1]. The Global Burden of disease project in 2017 reported that 1.42% of all deaths in Iran were due to cirrhosis and liver-related disorders [2]. Cirrhosis is defined by progressive liver damage resulting in transformed lobular architecture and lowered functional capacity [3]. Infections, hepatitis B and C, non-alcoholic fatty liver disease (NAFLD), metabolic syndrome and alcohol are etiologic factors of cirrhosis [4]. Cirrhosis complications are various from no symptoms in the compensated state to nausea, ascites and loss of appetite in mild cirrhosis and sarcopenia, spontaneous bacterial peritonitis, variceal bleeding, renal failure and hepatic encephalopathy in advanced stages [5].

Dietary therapy plays a major role in reversing the progression of liver cirrhosis and nutritional evaluation aims to reduce morbidity and mortality by exploring modifiable nutritional risk factors [6]. The liver is the central core of fat, protein and carbohydrate metabolism, thus it is predictable that liver cirrhosis may result in protein energy malnutrition (PEM) [7]. PEM is directly related to survival in cirrhosis and its prevalence varies from relatively 60% in decompensated and 20% in compensated cirrhotic patients [8]. Protein sources such as animal, dairy and vegetable may have different impacts on cirrhosis and are recommended to prevent sarcopenia and improve nitrogen balance [9].

According to the European Society of Clinical Nutrition and Metabolism (ESPEN), the daily recommended intake of protein in cirrhotic patient is 1.2 to 1.5 g/kg body weight [9]. Reduced skeletal muscle volume and plasma albumin level are the indicators of muscular and visceral protein loss, respectively [10]. Analyzing the plasma amino acids in cirrhosis indicates decreased branched-chain amino acids (BCAAs) and increased aromatic amino acids (AAAs) [7, 10]. BCAAs are used to detoxify ammonia and energy production [10]. Vegetables and dairy products are rich in BCAAs, which can improve physical and mental status, regulate protein imbalance, regenerate hepatic cells, increase albumin levels and reinforce immune system [10–12].

Considering previous studies, higher protein intake was associated with lower morbidity and all-cause mortality in various diseases [13–15]. However, little is known about dietary protein intake and cirrhosis-related mortality. In a current prospective cohort study, we aimed to evaluate the association between dietary total, animal, vegetable and dairy protein intake with cirrhosis-related mortality.

## Methods and materials

### Study population

In this cohort study, 166 ambulatory cirrhotic patients who had been diagnosed for at least 6 months were recruited from two educational hospitals, in Tehran, Iran. The participants were enrolled from 2016 to 2018 and were followed up until 30 April 2022. Exclusion criteria included the following: having renal failure, malignancies, diabetes mellitus, infectious diseases, cardiac disease, acquired immune deficiency syndrome, pancreatic insufficiency and being pregnant or lactating. Also, we excluded participants (n = 45) with a cancer diagnosis in the first year, missing or incomplete general lifestyle or dietary information, taking high or low energy (< 500 or > 5000 kcal/day) and extreme body mass index (BMI) (< 15 or > 50 kg/m^2^). In total, 121 participants (38 women and 83 men) remained for the analyses. Participants received annual telephone calls during which follow-up questionnaires were completed regarding the occurrence of death or any medical event.

The protocol of this study was approved by National nutrition and Food Technology Research Institute (NNFTRI) ethics committee (Ir.sbmu.nnftri.1396.186.). All participants were informed about the study and written consent forms were obtained.

### Dietary assessment

Dietary intakes of participants were collected using a reliable and valid food frequency questionnaire (FFQ) including 168 items through face-to-face interview [16]. During the interview, trained dieticians explained serving and typical portion sizes for each food item to participants, and then asked about the number of times each item was taken in the past year. Monthly, weekly and daily consumption of each food was documented and converted to grams based on household measurements [17, 18]. We used Nutritionist IV software to analyze dietary data. Energy and nutrient content were calculated using The United States Department of Agriculture (USDA) food composition table (FCT). In addition to total protein, the intakes of dairy, vegetable and animal protein were calculated and reported as gram per day.

### Potential confounders

Participants’ data including age, gender, alcohol consumption, smoking, subjective global assessment tool (SGA), Child–Pugh score, Model for end-stage liver disease (MELD) and etiology of cirrhosis were collected. A digital scale to the nearest 100 g and a portable stadiometer to the nearest 1 cm were applied to measure the weight and height of the participants with minimal clothes and without shoes, respectively. BMI was calculated by dividing the weight in kilograms by the square of the height in meters. SGA based on Destky et al. study [19] was recorded. Considering this assessment, participants were divided into three groups: A: well-nourished, B: moderately malnourished and C: severely malnourished. Severity and prognosis of liver cirrhosis were evaluated by Child–Pugh and MELD scores [17]. Prothrombin time, serum bilirubin, serum albumin, presence of hepatic encephalopathy and ascites are used to calculate Child–Pugh score by which the patients were classified into three groups.

Muscle strength was assessed based on hand grip strength in the dominant hand using a hydraulic hand dynamometer (Exacta, North Coast Medical, Giliory, USA). Low muscle strength was considered as a hand grip strength less than 26 kg for men and less than 18 kg for women [20] and consequently dynapenia was determined [21].

### Statistical analysis

Participants were divided into three groups based on their dietary protein intake. Also, according to BMI and dynapenia, participants were assigned in one of the following four groups: dynapenic obese, nondynapenic obese, dynapenic non-obese and nondynapenic non-obese. Basic characteristics of patients were compared using chi-squared (χ2) test for categorical variables and one-way analysis of variance (ANOVA) test for continuous variables. We used Cox proportional hazards regression models for cirrhotic-related mortality associated with the dietary total, dairy, vegetable and animal protein intake to estimate 95% confidence intervals (CIs) and multivariable-adjusted hazard ratios (HRs). Potential confounders were defined in the following series: Model 1: adjusted for sex (male, female) and age (continuous); Model 2: additionally adjusted for BMI (continuous), energy intake (continuous), alcohol use (yes, no) and smoking (yes, no); and Model 3: additionally adjusted for Child–Pugh (A, B & C), MELD (continuous) and etiology (virus, autoimmune, other). In relation to risk of mortality, we used the likelihood ratio test (LRT) to assess potential interactions between important risk factors at baseline such as age, BMI, MELD, Child–Pugh and SGA and intake of total, dairy, animal and vegetable protein. From the date of participation until censoring on 30 April 2022 or lost to follow-up or date of death, whichever occurred first was considered as person-years of follow-up. Statistical analyses were conducted using SPSS (version 19; SPSS Inc, Chicago, IL, USA) and *P* < 0.05 was set as statically significant.

## Results

Characteristics of participants are shown in Table.1. At the baseline the mean age ± standard deviation (SD) of patients was 54.8 ± 11.9 years. Overall, 68% of participants were men and the etiology in 56% of cirrhotic patients was viral hepatitis. In 3955 person-month of follow-up, 43 deaths (36 men and 7 women) occurred. Liver failure was responsible for 47% of deaths, cardiovascular diseases 40%, carcinoma 3% and other causes for 10% of deaths. The mean of total energy and energy-adjusted protein intake of patients was 2123 kcal and 78 g, respectively. On average, intake of total, dairy, vegetable and animal protein was 27, 13 and 26 g per day, respectively. The mean BMI of participants was 27.1 kg/m^2^ and relatively half of patients (47%) were dynapenic. Smoking in 39% and alcohol intake in 23% of participants were reported.

**Table 1:** Characteristics of participants

Variable	Mean ± SD	%
Age	54.8 ± 11.9	
Male		68
Etiology of cirrhosis		
Virus		56
Autoimmune		31
Other		13
MELD score	12.2 ± 4.9	
Child Pugh category (A/B/C)		
A		69
B & C		31
Alcohol drinker		23
Smoker, %		39
Subjective global assessment (A/B/C)		
A		32
B		54
C		14
Dynapenia		47
Dry weight, kg	73.9 ± 16.4	
Height, cm	165.4 ± 8.3	
BMI, kg/m^2^	27.1 ± 5.3	
Calorie intake (Kcal/day)	2123 ± 649	
Protein intake (g/day)	78 ± 27	
Protein intake (% of energy)	14 ± 2	
Protein intake, g/kg estimated dry weight	1.2 ± 0.5	
< 0.8 g/kg (very low protein intake)		21
0.8–1.2 g/kg (low protein intake)		34
> 1.2 g/kg (target protein intake)		45
Dairy protein (g/day)	27 ± 24	
Vegetable protein (g/day)	13 ± 9	
Animal protein (g/day)	26 ± 16	

Values are means ± SDs for continuous variables and percentages for categorical variables

Characteristics of participants according to dynapenia and obesity classifications are shown in Table 2. Based on SGA, the prevalence of malnutrition was significantly higher in non dynapenic obese patients than in dynapenic non-obese patients. Also, the severity of the disease in dynapenic patients was more severe than non-dynapenic patients, based on MELD score. Comparison of non-obese patients showed that the mean age of those with dynapenia is significantly higher than non-dynapenic patients. According to Child Pugh score, dynapenia in obese patients was significantly associated with worse prognosis of patients with cirrhosis.

**Table 2:** Characteristics of participants classified according to dynapenia and obesity classifications

	Dynapenic obese	Nondynapenic obese	Dynapenic non-obese	Nondynapenic non-obese
Prevalence (%)	36	37	22	26
No. of deaths	19	6	10	8
Male, %	56	73	64	66
Age	56 ± 11	55 ± 9	62 ± 12	48 ± 13^‡^
MELD score	14 ± 6^†^	10 ± 3	14 ± 7^†^	12 ± 4
Child Pugh category		*		
A	42	85	60	74
B & C	58	15	40	26
Alcohol drinker, %	22	37	12†	25
Smoker, %	31	56	28	43
Subjective global assessment			†	
A	28	50	11	27
B	56	44	61	59
C	16	6	28	14
BMI, kg/m^2^	29 ± 4	31 ± 4	21 ± 1^†^*	22 ± 2^†^*
Calorie intake (Kcal/day)	1763 ± 576	2539 ± 586*	1835 ± 594†	2217 ± 682*

Values are means ± SDs for continuous variables and percentages for categorical variables

ANOVA for quantitative variables and χ2 test for qualitative variables

^*^P < 0.05 versus dynapenic obese

^†^*P* < 0.05 versus nondynapenic obese

^‡^*P* < 0.05 versus dynapenic non-obese

Obese: BMI >  = 25

Confidence intervals (CIs) and multivariable-adjusted hazard ratios (HRs) for cirrhotic-related mortality related to dietary total, animal, vegetable and dairy protein intake are shown in Table 3.

**Table 3:** Hazard ratios for total mortality, according to the protein intake tertile

	Tertiles of dietary protein intake (g/day)			*P* trend
**Total protein (g/day)**	T1 (< 63)	T2 (63–89)	T3 (89 <)	
No. of deaths	20	17	6	
Model 1	ref	0.93 (0.45–1.9)	0.33 (0.12–0.87)	0.031
Model 2	ref	0.88 (0.3–2.5)	0.45 (0.1–2.05)	0.028
Model 3	ref	0.78 (0.2–3.5)	0.38 (0.2–1.1)	0.045
	Tertiles of dietary protein intake (g/kg)			*P* trend
**Total protein (g/kg)**	T1 (< 0.9)	T2 (0.9–1.4)	T3 (1.4 <)	
No. of deaths	20	17	6	
Model 1	ref	0.48 (0.24–0.98)	0.37 (0.16–0.83)	0.002
Model 2	ref	0.5 (0.2–1.14)	0.32 (0.1–1)	0.021
Model 3	ref	0.68 (0.14–3)	0.45 (0.1–2.4)	0.003
**Dairy protein**	T1 (< 11)	T2 (11–32)	T3 (> 32)	
No. of deaths	20	13	10	
Model 1	ref	0.3 (0.13–0.69)	0.32 (0.14–0.7)	0.024
Model 2	ref	0.5 (0.2–1.4)	0.6 (0.2–1.7)	0.098
Model 3	ref	0.45 (0.2–1.04)	0.38 (0.13–1.1)	0.046
**Vegetable protein**	T1 (< 7)	T2 (7–16)	T3 (> 16)	
No. of deaths	20	12	11	
Model 1	ref	0.5 (0.23–1.05)	0.47 (0.2–1.03)	0.022
Model 2	ref	0.85 (0.3–2.4)	0.6 (0.2–1.5)	0.398
Model 3	ref	0.65 (0.2–1.8)	0.58 (0.13–2.6)	0.633
**Animal protein**	T1 (< 15)	T2 (15–33)	T3 (> 33)	
No. of deaths	10	15	18	
Model 1	ref	1.4 (0.6–3.2)	1.4 (0.6–3.3)	0.207
Model 2	ref	2.6 (0.9–6.8)	2.3 (0.9–5.9)	0.021
Model 3	ref	2.9 (1.4–5.9)	3.8 (1.7–8.2)	0.035

Cox proportional hazards regression models for estimating HRs and 95% Cis

Model 1: adjusted for age and sex

Model 2: additionally adjusted for energy intake, BMI, smoking, alcohol

Model 3: additionally adjusted for etiology, MELD and child

After adjusting the results for age and sex, intake of total protein, dairy and vegetable protein was significantly associated with a reduction in the risk of mortality in cirrhotic patients. In this model, protein intake was associated with a non-significant increase in mortality risk. By further adjusting the results for energy intake, BMI, smoking and alcohol, this significance was lost for vegetable and dairy protein, but the association of animal protein intake with increased risk of mortality in cirrhosis patients became significant.

As shown in Table 3, after adjusting for all confounders, it has been found that the mortality risk of participants in the third tertile of total dietary protein intake was significantly lower than that of the first tertile (HR _T3 vs T1_ = 0.38, 95% CI = 0.2–1.1, P trend = 0.045). The statistical analysis of total protein, both in grams per day and in grams per weight, had similar results. Similar results were obtained for dairy protein intake. So that the risk of mortality in the third tertile was 62% lower compared to the reference group (HR _T3 vs T1_ = 0.38, 95% CI = 0.13–1.1, P trend = 0.046). It is noteworthy that higher intake of animal protein was associated with increased risk of mortality (HR _T3 vs T1_ = 3.8, 95% CI = 1.7–8.2, P trend = 0.035). Higher intakes of vegetable protein were inversely but non-significantly associated with risk of mortality.

**Fig. 1:**
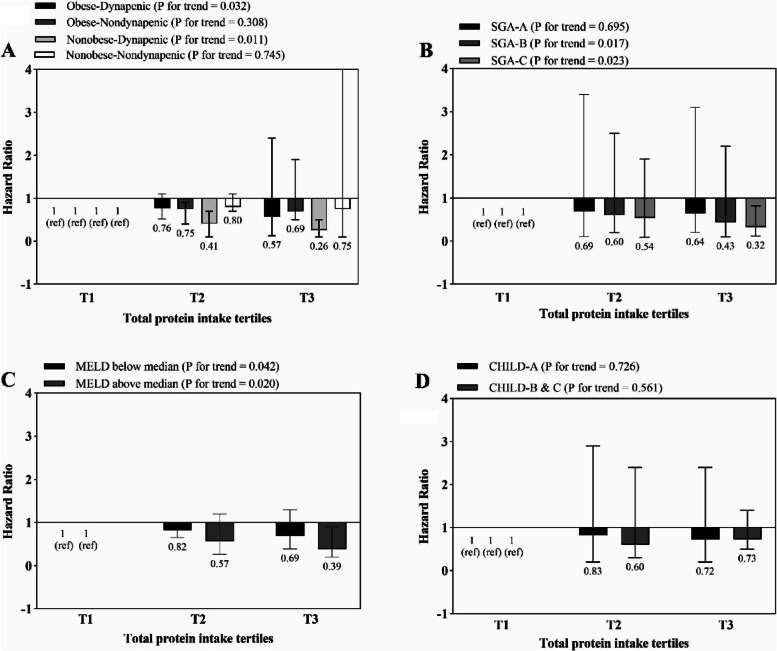
Multivariate hazard ratios of total protein intake tertiles for cirrhosis-related mortality according to risk factor status at baseline (Cox proportional hazards regression models for estimating HRs and 95% CIs, multivariable models were adjusted for sex, age, energy intake, BMI, smoking, alcohol, etiology, MELD and child, except for the respective stratifying factor). Data are reported as HR (95% CI). **A** obesity-dynapenia phenotypes (*P* = 0.026 for interaction); **B** SGA A vs B and C (*P* = 0.056 for interaction); **C** MELD score below median vs above median (*P* = 0.239 for interaction); **D** Child Pugh A vs B&C (*P* = 0.637 for interaction). Ref indicates reference group. BMI: body mass index, subjective global assessment tool (SGA), Model for end-stage liver disease (MELD)

**Fig. 2:**
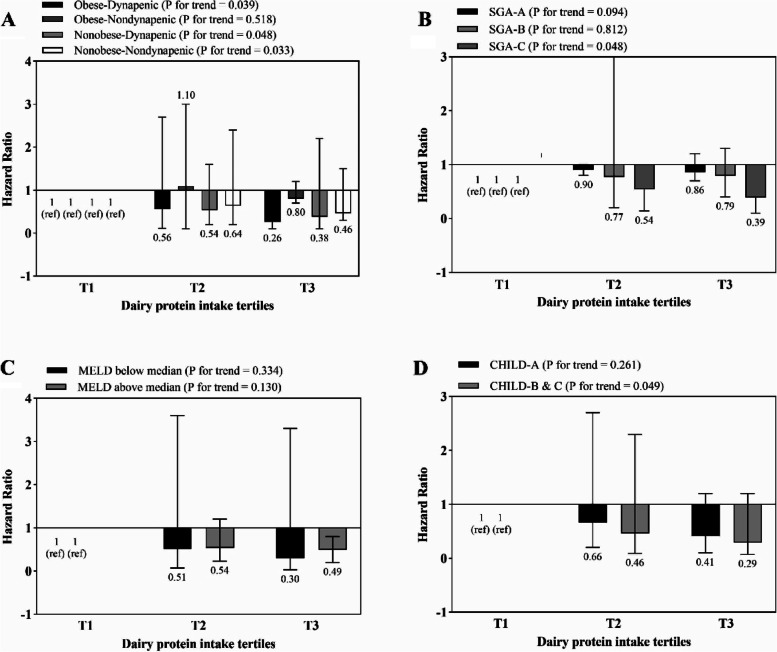
Multivariate hazard ratios of dairy protein intake tertiles for cirrhosis-related mortality according to risk factor status at baseline (Cox proportional hazards regression models for estimating HRs and 95% CIs, multivariable models were adjusted for sex, age, energy intake, BMI, smoking, alcohol, etiology, MELD and child, except for the respective stratifying factor). Data are reported as HR (95% CI). **A** obesity-dynapenia phenotypes (*P* = 0.003 for interaction); **B** SGA A vs B and C (*P* = 0.767 for interaction); **C** MELD score below median vs above median (*P* = 0.165 for interaction); **D** Child Pugh A vs B&C (*P* = 0.362 for interaction). Ref indicates reference group. BMI: body mass index, subjective global assessment tool (SGA), Model for end-stage liver disease (MELD)

**Fig. 3:**
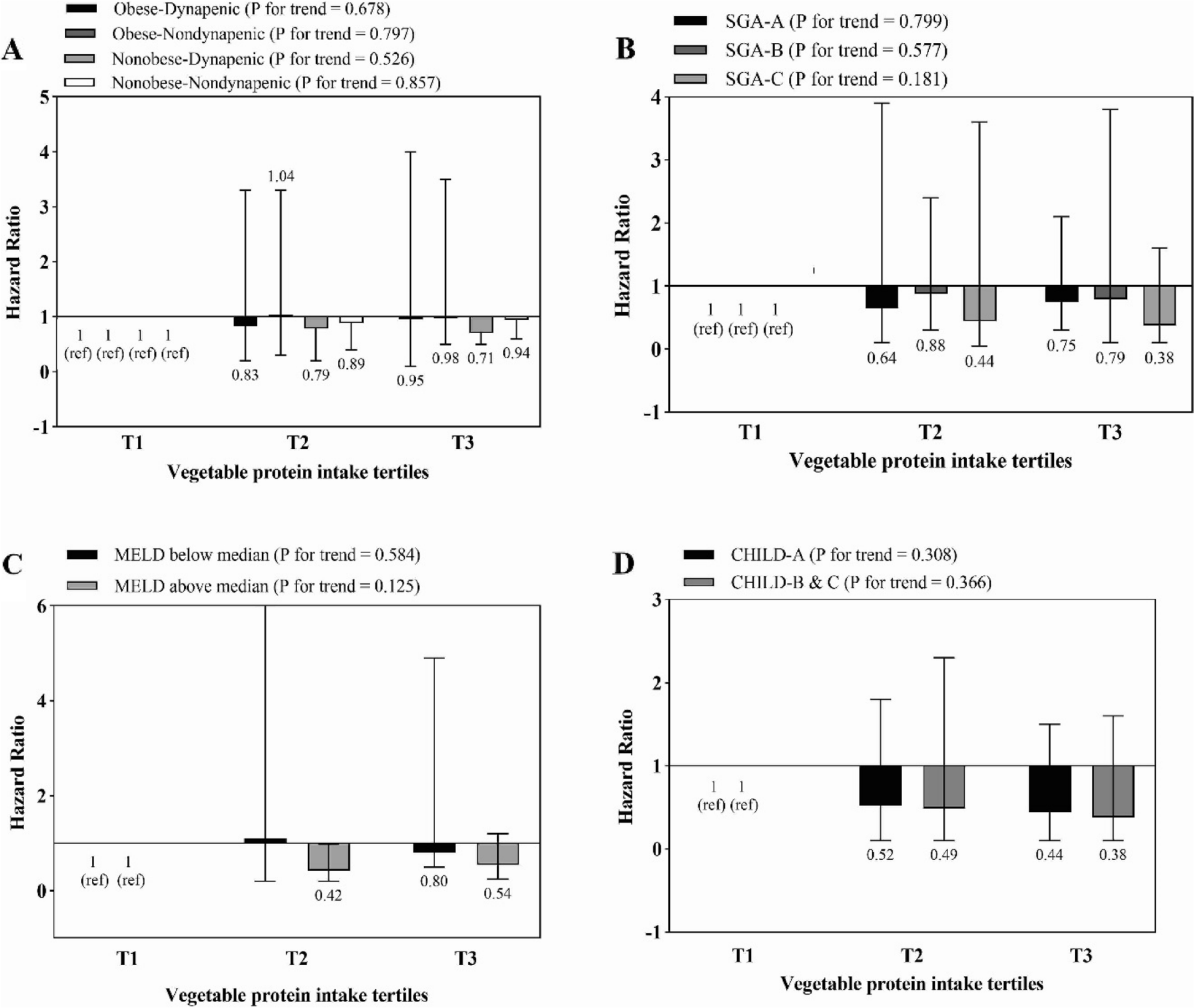
Multivariate hazard ratios of vegetable protein intake tertiles for cirrhosis-related mortality according to risk factor status at baseline (Cox proportional hazards regression models for estimating HRs and 95% CIs, multivariable models were adjusted for sex, age, energy intake, BMI, smoking, alcohol, etiology, MELD and child, except for the respective stratifying factor). Data are reported as HR (95% CI). **A** obesity-dynapenia phenotypes (*P* = 0.026 for interaction); **B** SGA A vs B and C (*P* = 0.056 for interaction); **C** MELD score below median vs above median (*P* = 0.239 for interaction); **D** Child Pugh A vs B&C (*P* = 0.637 for interaction). Ref indicates reference group. BMI: body mass index, subjective global assessment tool (SGA), Model for end-stage liver disease (MELD)

**Fig. 4:**
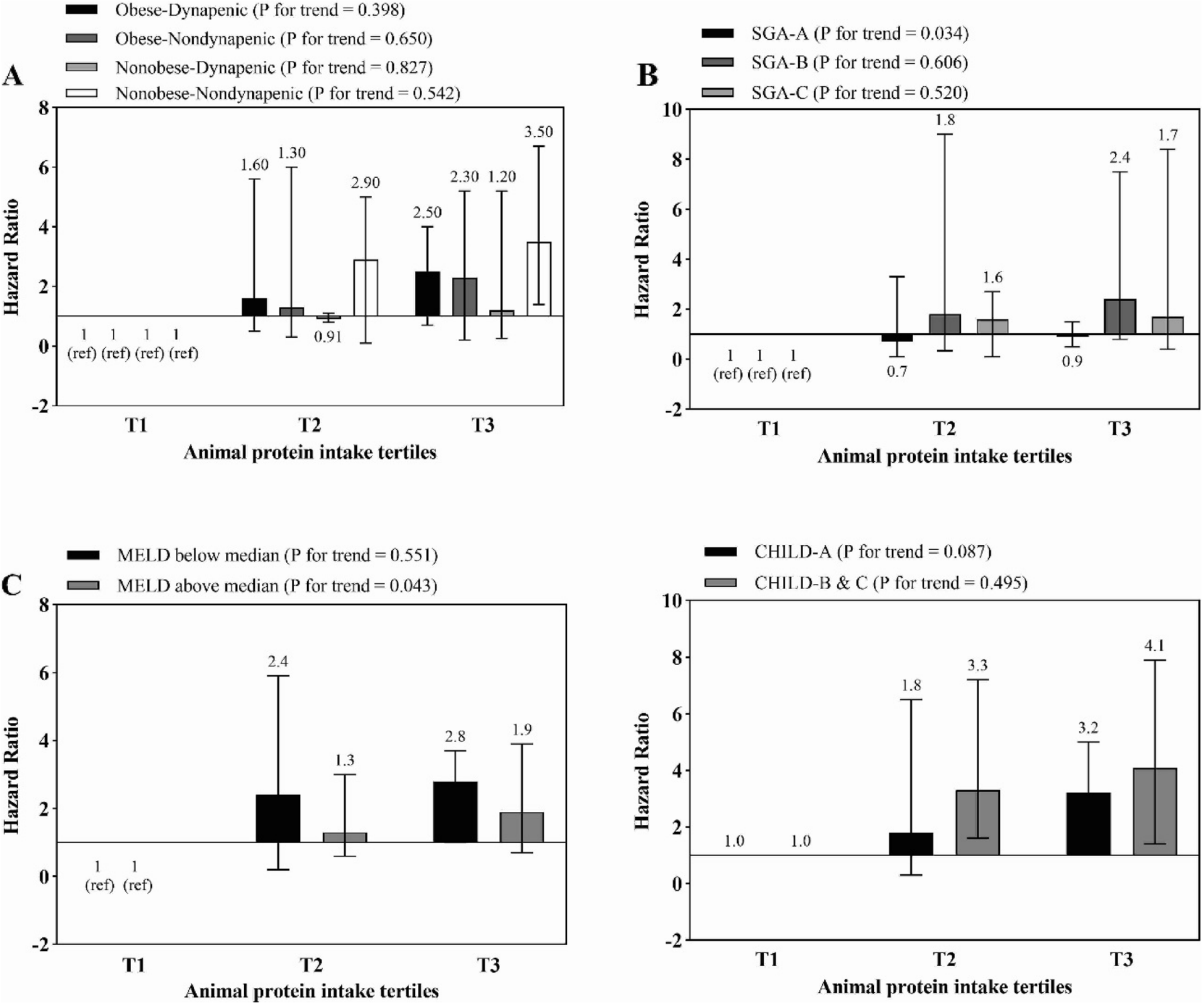
Multivariate hazard ratios of animal protein intake tertiles for cirrhosis-related mortality according to risk factor status at baseline (Cox proportional hazards regression models for estimating HRs and 95% CIs, multivariable models were adjusted for sex, age, energy intake, BMI, smoking, alcohol, etiology, MELD and child, except for the respective stratifying factor). Data are reported as HR (95% CI). **A** obesity-dynapenia phenotypes (*P* = 0.592 for interaction); **B** SGA A vs B and C (*P* = 0.363 for interaction); **C** MELD score below median vs above median (*P* = 0.079 for interaction); **D** Child Pugh A vs B&C (*P* = 0.781 for interaction). Ref indicates reference group. BMI: body mass index, subjective global assessment tool (SGA), Model for end-stage liver disease (MELD)

The association between total, dairy, vegetable and animal protein intake and risk of mortality is shown in Figs. 1, 2, 3 and 4, respectively. Higher intake of total and dairy protein significantly decreased the risk of mortality in dynapenic and severely malnourished patients. In addition, in patients with higher disease severity (MELD score above the median > 11), higher animal protein intake was significantly associated with an increased risk of mortality (HR _T3 vs T1_ = 1.9, 95% CI = 0.7–3.9, P trend = 0.043) (Fig. 4).

**Fig. 5:**
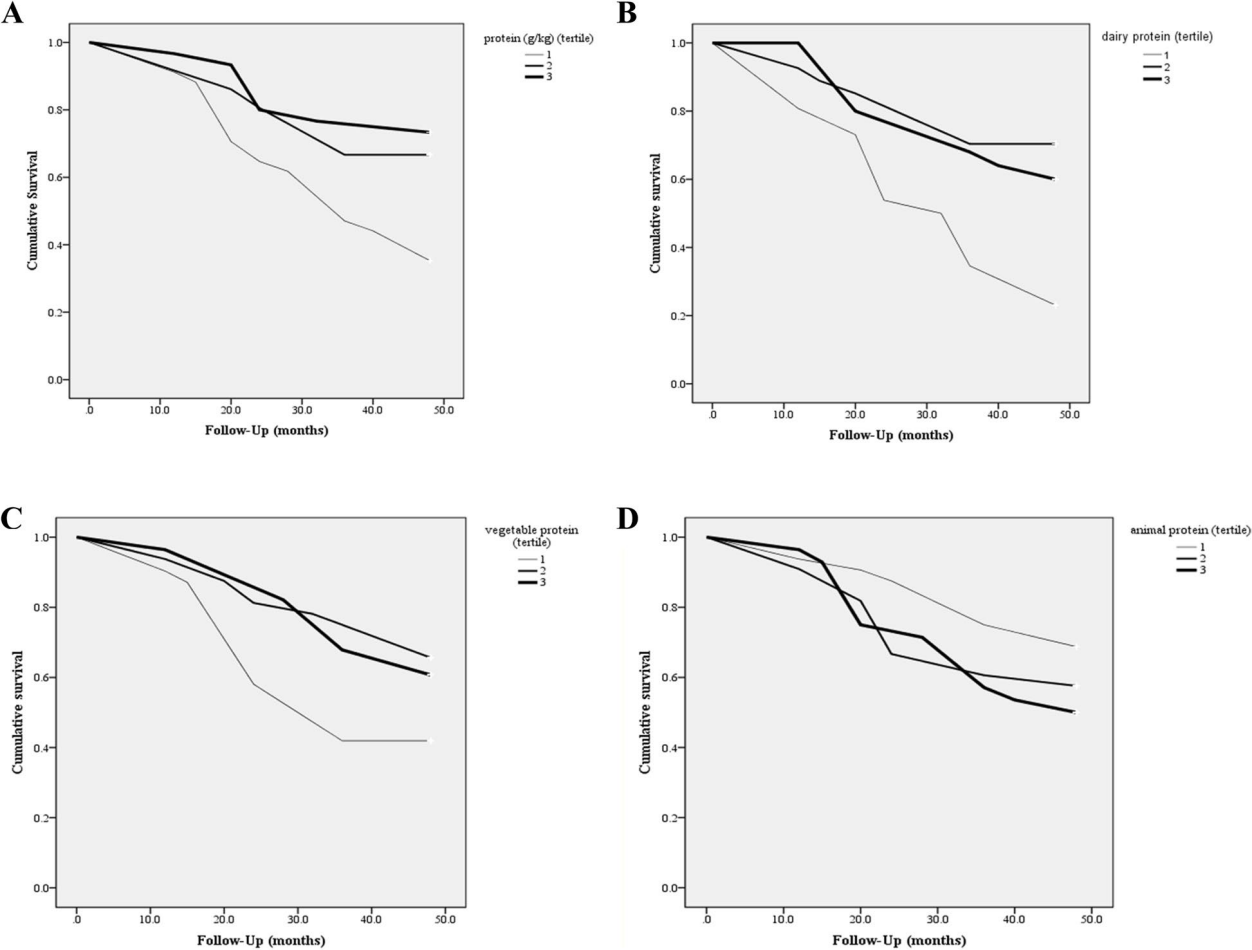
**A** Kaplan–Meier survival curve for death among cirrhotic patients stratified by tertiles of dietary protein intake (grams per kilogram body weight). The 4-year survival rate among patients across tertiles was 35%, 40%, 43%, respectively (log-rank test for homogeneity, *P* = 0.004). **B** Kaplan–Meier survival curve for death among cirrhotic patients stratified by tertiles of diary protein intake (grams per day). The 4-year survival rate among patients across tertiles was 32%, 40%, 43%, respectively (log-rank test for homogeneity, *P* = 0.001). **C** Kaplan–Meier survival curve for death among cirrhotic patients stratified by tertiles of vegetable protein intake (grams per day). The 4-year survival rate among patients across tertiles was 35%, 42%, 41%, respectively (log-rank test for homogeneity, *P* = 0.072). **D** Kaplan–Meier survival curve for death among cirrhotic patients stratified by tertiles of animal protein intake (grams per day). The 4-year survival rate among patients across tertiles was 42%, 38%, 37%, respectively (log-rank test for homogeneity, *P* = 0.296)

The Kaplan–Meier survival curves comparing patients across tertiles of dietary protein intake are shown in Fig. 5 (A: total protein, B: dairy protein, C: vegetable protein, D: animal protein). Patients with lower dairy protein (T1) had significantly worse 4-year survival compared with patients with higher dairy protein. Comparison of the third tertile with the first tertile of total protein and vegetable protein intake also revealed a similar result, but it was not statistically significant. Higher animal protein intake was associated with worse 4-year survival, but not significantly.

## Discussion

The present cohort study showed that higher intake of dietary total protein and dairy protein and lower intake of animal protein was associated with reduced risk of mortality in cirrhotic patients, after full adjustment of confounding factors such as sex, age, smoking, alcohol, BMI, energy intake, etiology, Child Pugh and MELD score.

Consistent with the findings of the present study, previous studies have shown the relationship between dietary total and vegetable protein intake and the reduction of the risk of mortality [13, 15], as well as the relationship between dietary animal protein intake and the increase of the risk of mortality [15] in various diseases. Also, replacing animal protein, especially processed red meat, with vegetable protein reduced the risk of mortality, which indicates the importance of the protein source [22]. An evaluation of a large cohort study in the United States of men and women with 16 years of follow-up presented that higher intake of plant protein decreased the risk of CVD and all-cause mortality in both sexes. Also, in this study, a significant inverse relationship was observed in replacing red meat and egg protein with vegetable protein, including pasta, bread, and grains [23].

Vegetable protein may reduce systolic and diastolic blood pressure, improve lipoprotein and lipid profile and decrease insulin-like growth factor-1(IGF-1) [24–26]. In agreement with our study, in a large cohort study on cirrhotic patients waiting for liver transplant, it has been reported that low protein intake was prevalent and resulted in liver disease severity and worse clinical outcomes [27]. Also, it was independently associated with mortality and malnutrition [27]. Sam et al. reported that protein-energy malnutrition was prevalent in cirrhotic patients and was associated with higher in-hospital mortality [28]. Moreover, in another study, it has been shown that in cirrhotic conditions, restricted dietary protein may stimulate protein catabolism and worsen hepatic encephalopathy [29]. In cirrhotic patients, protein requirement is higher than healthy population due to alterations in protein metabolism, PEM, muscle breakdown and protein-losing enteropathy caused by portal hypertension which may result in excessive intestinal protein losses [30]. Several factors connected low protein intake with cirrhosis-related mortality. First, protein is an important substance in human body necessary for carrying out vital body functions. Second, infection is a major cause of death in cirrhotic patients and low protein diet impairs both humoral and cell-mediated immunity and low dietary protein causes pro-inflammatory state [31]. Third, sufficient protein intake is necessary for muscle synthesis. In cirrhotic patients, glycogen reserves and synthetic capacity of liver is impaired, compensated by increased gluconeogenesis using amino acids of skeletal muscles [32].

The relationship between the dairy and vegetable protein intake and better nutritional and clinical status of cirrhotic patients has been already demonstrated [33]. Consistently, in a randomized cross-over trial, Bianchi et al. reported that in cirrhotic patients with chronic encephalopathy, vegetable protein could ameliorate nitrogen balance and mental state [34]. In addition to protein, vegetables are also rich in fiber. By inducing the mass of colon bacteria, fiber increases nitrogen consumption and reduces the incidence of hepatic encephalopathy [35].

Furthermore, the low level of plasma BCAAs including valine, leucine and isoleucine, is hallmark feature of cirrhosis patients [36]. Dairy and vegetable products are rich sources of BCAAs. Ruiz-Margain et al. reported that high-fiber, high-protein diet and BCAAs might increase muscle mass and prevent the increase in glucose and ammonium levels [37]. Another randomized control trial on cirrhotic patients showed that BCAAs supplementation improved prognosis, quality of life, nutritional status and albumin synthesis [12]. BCAAs activate mTOR signaling pathway which stimulates synthesis of muscle protein and albumin and regenerates liver cells [36].

Investigating the association of protein intake, separated by source, with the risk of cirrhosis-related mortality for the first time is one of the strengths of a current cohort study. In addition, adjusting potential confounders increased the reliability of the present study. The present study has limitations that should be considered, including a relatively small study population, inevitable recall bias using food frequency questionnaire (FFQ) and missing of about 15% of enrolled patients.

## Conclusion

In conclusion, we found a significant reverse association between total and dairy protein intake and a significant direct association between animal protein intakes with cirrhosis-related mortality. Further studies are recommended to evaluate effectiveness and appropriate amount of total, dairy, vegetable and animal protein in cirrhotic patients.

## Data Availability

The datasets analyzed in the current study are available from the corresponding author on reasonable request.

## Abbreviations

HR: Hazard ratio
CIs: Confidence intervals
NAFLD: Non-alcoholic fatty liver disease
PEM: Protein energy malnutrition
ESPEN: European Society of Clinical Nutrition and Metabolism
BCAA: Branched chain amino acids
NNFTRI: National nutrition and Food Technology Research Institute
FFQ: Food frequency questionnaire
USDA: The United States Department of Agriculture
FCT: Food composition table
SGA: Subjective global assessment tool
MELD: Model for end-stage liver disease
ANOVA: One-way analysis of variance
BMI: Body mass index
LTR: Likelihood ratio test
SPSS: Statistical Package Software for Social Science
IGF-1: Insulin-like growth factor-1
